# Decision-Aware Vision Mamba with Context-Guided Slot Mixing for Chest X-Ray Screening and Culture-Based Hierarchical Tuberculosis Classification

**DOI:** 10.3390/s26072100

**Published:** 2026-03-27

**Authors:** Wangsu Jeon, Hyeonung Jang, Hongchang Lee, Chanho Park, Jiwon Lyu, Seongjun Choi

**Affiliations:** 1 Department Computer Engineering, Kyungnam University, Changwon 51767, Republic of Korea; jws2218@naver.com; 2 Gyungnam Intelligence Innovation Center, Kyungnam University, Changwon 51767, Republic of Korea; 3 Haewootech Co., Ltd., Busan 46742, Republic of Korea; hwjang@haewootech.co.kr (H.J.); hclee@haewootech.co.kr (H.L.); 4 Department of Radiology, Soonchunhyang University Cheonan Hospital, Cheonan 31151, Republic of Korea; 98480@schmc.ac.kr; 5 Division of Respiratory Medicine, Department of Internal Medicine, Soonchunhyang University Cheonan Hospital, Cheonan 31151, Republic of Korea; 6 Department of Otolaryngology-Head and Neck Surgery, Cheonan Hospital, Soonchunhyang University College of Medicine, Cheonan 31151, Republic of Korea; 7 MDAI Co., Ltd., Cheonan 31151, Republic of Korea

**Keywords:** Tuberculosis, chest X-ray, State Space Models, Vision Mamba, active TB, inactive TB, explainable AI

## Abstract

Distinguishing Active from Inactive Tuberculosis (TB) on Chest X-rays presents a clinical challenge due to overlapping radiological signs. This study introduces Vision Mamba CGSM, a deep learning framework integrating a State Space Model (SSM) backbone with a Context-Guided Slot Mixing (CGSM) module. The SSM captures global anatomical context, while the CGSM module isolates subtle pathological features by applying localized spatial attention. We validated the model using a hierarchical diagnostic scheme covering Normal, Pneumonia, Active TB, and Inactive TB. Experimental evaluations demonstrate an accuracy of 92.96% and a Youden Index of 79.55% on the independent test set. In the specific binary classification of Active vs. Inactive TB, the model recorded a specificity of 97.04%, outperforming standard baseline architectures including ResNet152 and ViT-B. Additional validations on external datasets confirm the consistent generalization of the proposed feature extraction mechanism.

## 1. Introduction

Tuberculosis (TB) remains one of the world’s deadliest infectious diseases, causing approximately 1.08 million deaths in 2024 among HIV-negative individuals, making it a significant global health challenge that requires early and accurate diagnosis for effective treatment and transmission reduction [1,2]. A critical aspect of TB management is distinguishing between active and inactive forms of the disease. Rim et al. [3] demonstrated that active TB can be effectively distinguished from inactive TB using deep learning approaches combining EfficientNet-B7 with MLP-Mixer architectures. Choi et al. [4] further validated this approach, achieving 0.980 AUC on internal validation for TB activity determination, performing comparably to expert radiologists. This distinction is clinically essential because inappropriate treatment of inactive TB leads to unnecessary medication side effects and healthcare costs, while missed diagnosis of active TB results in disease transmission and mortality.

Recent comparative studies have provided insights into optimal model selection for TB classification. Mirugwe et al. [5] systematically compared six CNN architectures, finding that simpler models like VGG16 achieved 97.4% accuracy with 11× fewer parameters than ResNet152, demonstrating that architectural complexity does not always translate to better performance. Complementing this analysis, Kotei and Thirunavukarasu [6] identified VGG, ResNet, and DenseNet as the predominant architectures in this field, while also noting persistent challenges in model interpretability and generalization across datasets.

Several recent studies have contributed to practical implementations for TB detection. Shekar and Mannan [7] developed a three-layer CNN with oversampling techniques to address class imbalance in TB datasets. Abraham et al. [8] proposed a CNN combined with PatternNet classifier for computer-aided TB detection from X-ray images. García Seco de Herrera et al. [9] developed ensemble architectures incorporating Convolutional Autoencoder and Multi-Scale CNNs, achieving 99% sensitivity and 94% specificity on the Shenzhen dataset.

Interpretability and visualization have emerged as critical components of clinical TB detection systems. Sharma et al. [10] achieved 99.29% accuracy with 99.9% AUC, with heatmaps consistently highlighting upper lung lesions characteristic of active TB. Chen et al. [11] developed deep learning algorithms utilizing accessible computational tools suitable for resource-limited clinical settings. Visu et al. [12] achieved 99.06% accuracy using Enhanced Swin Transformer with U-Net segmentation, demonstrating the potential of modern transformer-based approaches. Nafisah and Muhammad [13] combined CNNs with explainable AI techniques, showing that models correctly attend to upper lung zones and cavities aligned with expert radiological assessment.

Advanced diagnostic approaches have extended beyond binary TB detection. Devasia et al. [14] developed multi-label classification models identifying TB manifestations in specific lung zones (upper, middle, lower lobes), achieving zone-wise AUCs of 93–97% using EfficientNet-B4 with Grad-CAM visualization. Early deep learning research established the feasibility of automated chest X-ray analysis for TB. Lakhani and Sundaram [15] achieved 99% AUC using AlexNet and GoogleNet [16], demonstrating that CNNs could match radiologist performance for binary TB detection. Hwang et al. [17] developed algorithms that outperformed physicians with 97.3% sensitivity and 99.8% specificity on over 10,000 patients, demonstrating clinical viability of AI-assisted screening. Rahman et al. [18] showed that lung segmentation preprocessing significantly improved classification accuracy to 99.9% from 97.1% for whole images, confirming that CNNs benefit from focusing on relevant anatomical regions.

Recent comprehensive reviews have synthesized the state of TB detection research. Ait Nasser and Akhloufi [19] reviewed recent advances in deep learning for chest disease detection, highlighting the need for models that can handle multiple diseases and provide explainable predictions. Maheswari et al. [20] developed explainable shallow CNN architectures using Class Activation Mapping (CAM) [21] and LIME [22], achieving 95% accuracy with transparent decision rationale that clinicians could validate. Selvaraju et al. [23] introduced Grad-CAM, which became the standard technique for visualizing which regions of chest X-rays CNNs use for predictions. Sarawagi et al. [24] further validated the clinical utility of CNN-based diagnosis systems through self-trained approaches.

Standard diagnostic methods for TB include bacteriological tests such as sputum smear microscopy and culture, as well as molecular diagnostics like sputum MTB PCR and Xpert assays [25,26]. While molecular tests have improved diagnostic speed and accuracy, these methods collectively face limitations including low sensitivity in smear tests (30–40% false negative rates), long turnaround times for cultures, resource intensity for molecular assays, and a shared dependence on patient cooperation for quality sputum samples [1]. Chest X-ray (CXR) imaging serves as a first-line screening tool due to its availability, low cost, and rapid results [3], though radiological interpretation of TB activity status remains challenging and highly dependent on radiologist expertise [4].

Most existing deep learning research has focused on TB vs. non-TB classification rather than the clinically important distinction between active and inactive disease [3,4,6]. The limited research on active/inactive TB classification represents a significant gap in the literature, as this more detailed task requires models capable of recognizing subtle radiological differences such as cavity characteristics, nodule density, and consolidation vs. fibrotic changes [19].

Despite significant progress in computer-aided diagnosis, current deep learning architectures face limitations in capturing the subtle radiological patterns of TB. Convolutional Neural Networks (CNNs) primarily focus on local features, often failing to model long-range dependencies necessary for identifying diffuse or subtle lesions. While Vision Transformers (ViTs) address this through self-attention mechanisms, their quadratic computational complexity restricts efficiency, particularly with high-resolution chest X-rays. To address these challenges, we propose a novel framework employing the Vision Mamba architecture, which utilizes State Space Models (SSMs) to provide global receptive fields with linear computational complexity. We further integrate a Context-Guided Slot Mixing (CGSM) module designed to iteratively refine feature representations, enabling precise hierarchical classification of active and inactive TB based on microbiological culture confirmation.

## 2. Related Works

### 2.1. Deep Learning for Medical Imaging and Datasets

The application of deep learning to medical imaging has been revolutionized by foundational CNN architectures. Krizhevsky et al. [27] demonstrated breakthrough performance with AlexNet on large-scale image classification, sparking widespread adoption in medical imaging. Simonyan and Zisserman [28] introduced VGGNet, showing that network depth considerably impacts performance using small 3 × 3 convolutional filters in 16~19 layers architectures. He et al. [29] introduced ResNet with residual connections enabling training of networks up to 152 layers by addressing degradation problems. Huang et al. [30] further improved feature propagation through dense connections in DenseNet, while Tan and Le [31] introduced EfficientNet with compound scaling methods achieving better efficiency-accuracy tradeoffs. These architectures have become standard baselines for medical image classification [5,6,19].

Pham et al. [32] showed that hierarchical disease dependencies can be exploited during training to improve classification of overlapping pathologies in chest radiographs. Ronneberger et al. [33] introduced U-Net, which became the standard for medical image segmentation through encoder–decoder architecture with skip connections, widely used for lung region extraction and anatomical segmentation tasks [12,18]. Tolstikhin et al. [34] demonstrated that pure multi-layer perceptron architectures (MLP-Mixer) can achieve competitive performance, successfully applied to TB classification [3]. Dosovitskiy et al. [35] introduced Vision Transformers, showing that transformer architectures can match CNN performance by treating images as sequences of patches.

Jaeger et al. [36] contributed crucial public datasets that enabled reproducible research in the TB detection field. These datasets have become the standard benchmarks for evaluating TB detection algorithms. Standard data augmentation techniques including rotation, flipping, scaling, and brightness adjustment have become common practice for improving model performance on these limited datasets [5,18].

### 2.2. Active TB and Inactive TB Classification

Lee et al. [37] demonstrated that temporal changes in follow-up imaging could be automatically tracked for treatment response assessment, enabling longitudinal disease monitoring of TB activity. This work extended the capabilities of deep learning beyond single time point classification to dynamic assessment of disease progression.

Rahman et al. [38] developed TB-CXRNet for detecting both active TB and drug-resistant TB, addressing the critical challenge of identifying multidrug-resistant (MDR-TB) and extensively drug-resistant (XDR-TB) cases that require modified treatment protocols. Drug-resistant TB detection is essential given rising resistance rates globally.

Kazemzadeh et al. [39] developed a large-scale deep learning system trained on over 165,000 chest radiographs from 10 countries, demonstrating performance matching or exceeding that of radiologists in active TB detection, with an AUC of 89% and sensitivity of 88%. The system further projected 40–80% cost reductions in nucleic acid amplification test (NAAT)-based confirmatory testing workflows, establishing its suitability for mass screening programs in high-burden settings.

Owda et al. [40] addressed efficiency challenges by proposing a lightweight hybrid architecture combining GhostNet and MobileViT, which integrates spatial feature extraction with global contextual modeling to achieve competitive diagnostic accuracy while maintaining low computational overhead, making deployment feasible in resource-constrained settings. Grad-CAM-based visualization was further incorporated to provide radiologically interpretable predictions.

Alshmrani et al. [41] developed multi-class models distinguishing TB from other respiratory diseases including COVID-19 and pneumonia, achieving 93–96% accuracy across disease categories. This multi-disease classification capability is valuable for differential diagnosis in clinical settings.

### 2.3. Feature Localization and Preprocessing Techniques

Several studies have focused on refining feature localization for automated TB detection. Ejiyi et al. [42] introduced ResfEANet, a model that combines ResNet with external attention mechanisms to enhance both feature extraction and interpretability. This attention-based framework helps the model identify relevant diagnostic regions more effectively. Complementing these architectural improvements, Ou et al. [43] established that systematic preprocessing combined with U-Net variant architectures including Attention U-Net and U-Net++ is essential for guiding the model toward pulmonary regions of interest, demonstrating that ensemble segmentation models achieve superior TB lesion delineation with a mean IoU of 0.70 and F1-score of 0.81 compared to single-network approaches. A key technique in this process, as shown by Rahman et al. [18], is lung segmentation, which serves to reduce noise from anatomical structures such as the cardiac silhouette, mediastinum, and chest wall.

## 3. Proposed Method

We propose a novel deep learning framework, Vision Mamba CGSM, designed to hierarchically classify active and inactive TB with high precision. In the overall architecture, SSM is used to model global context [44,45] and a CGSM is implemented to refine feature representation iteratively. The proposed framework allows for the detection of subtle radiological signs, such as cavities or consolidations, by gradually focusing on clinically relevant regions.

### 3.1. Vision Mamba

**Figure 1:**
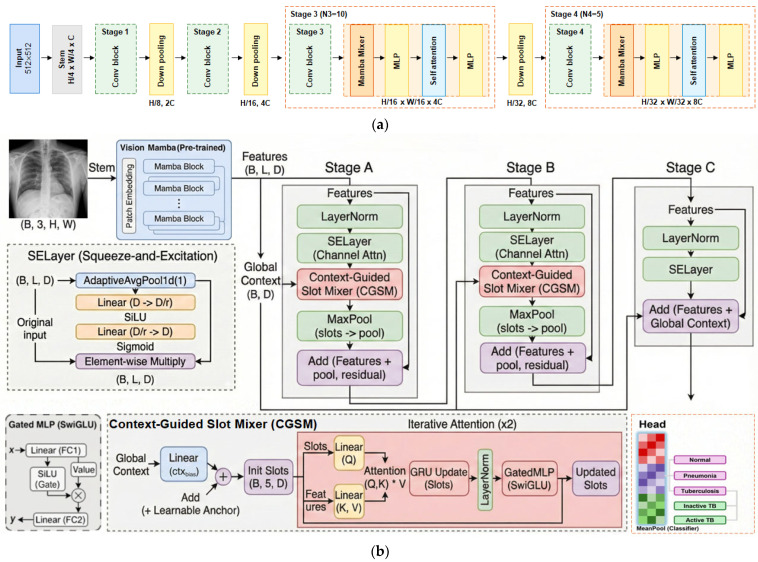
The overall proposed model. (**a**) Vision Mamba Backbone: Four Mamba-Transformer stages are used to extract multi-scale features through a hierarchical architecture. (**b**) Vision Mamba-CGSM: Proposed module that refines features through a systematic pipeline across three refinement stages (denoted as Stage A, B, and C), which process the features extracted from the backbone’s final stage. In this module, SELayer performs channel-wise recalibration while the CGSM extracts salient pathological information. The CGSM incorporates Global Context ($ctx_{bias}$) to stabilize slot initialization and performs iterative updates to enhance diagnostic features for final multi-label classification.

The input chest X-ray image X, with dimensions H × W × 3, is first processed by the Vision Mamba backbone [45], as illustrated in Figure 1a. The backbone processes the image patches and extracts hierarchical feature representations, producing a final flattened feature map F with dimensions B × L × D. Given the input resolution of 512 × 512 and the backbone’s total downsampling factor of 32, the resulting feature map F has a spatial resolution of 16 × 16, corresponding to a sequence length L of 256, with an embedding dimension of D = 1024. This feature map serves as the high-dimensional input for the subsequent refinement stages.

This architecture is distinct from traditional CNNs, which rely on local receptive fields, or ViTs, which suffer from quadratic computational complexity. Instead, Vision Mamba utilizes State Space Models (SSMs) [46] to model long-range dependencies with linear complexity relative to the sequence length. This capability is crucial for processing high-resolution medical images where diffuse lesions may span across large areas, ensuring efficient and global context modeling.

### 3.2. Context-Guided Slot Mixer (CGSM)

To enhance the localization of TB-specific lesions, we introduce the CGSM, inspired by the object-centric learning paradigm of Slot Attention [47]. This module creates a set of learnable vectors, called “slots,” which iteratively attend to and aggregate information from the feature map F. The operation of the CGSM consists of two key phases: context-aware initialization and iterative slot attention.

#### 3.2.1. Context-Aware Initialization

The initialization of slots by standard slot attention mechanisms involves randomly drawn noise from a Gaussian distribution. While this approach allows for maximum entropy during the initial training phase, it implies that the model has no prior knowledge of the image content. In chest X-ray analysis, inefficient use of initialization bias is possible due to the consistent anatomical structure of medical images, including the position of the spine, ribs, and lung fields. A context-aware initialization strategy is utilized to overcome this limitation and accelerate convergence by leveraging the global information of the input image to guide the slot generation process from the very beginning.

The process begins by extracting a global context vector from the feature map F generated by the Vision Mamba backbone. The input feature map has dimensions of B × L × D. We apply a Global Average Pooling (GAP) operation across the spatial dimension L, resulting in a global context vector g with dimensions B × D. As defined in Equation (1), this operation computes the mean across the spatial dimension:

(1) $$g=\frac{1}{L}\sum F_{i}$$

This vector summarizes the overall semantic content of the image. This global vector is then projected via a learnable linear weight matrix $W_{ctx}$ to produce a context bias term, ${ctx}_{bias}$, formulated in Equation (2):

(2) $${ctx}_{bias}=gW_{ctx}$$

We also define a set of learnable anchor slots, denoted as μ, with dimensions 1 × N × D. The final initial slot representation, S_0_ with dimensions B × N × D, is formed by combining these static anchors, the image-specific context bias, and a small random noise ε scaled by a variance parameter σ, as expressed in Equation (3):

(3) $$S_{0}=\mu +{ctx}_{bias}+(\sigma ⊗\varepsilon )$$

This hybrid initialization ensures that the slots are not generic but are conditioned on the specific anatomical context of the input patient scan. In our implementation, we set the number of slots N to 5. While this value is supported by our prior experimental findings on optimal feature disentanglement [48], our design choice is fundamentally motivated by the anatomical fact that human lungs are divided into five distinct lobes [49]. By explicitly aligning the number of slots with these biological regions, we aim to introduce a strong inductive bias that encourages the model to attend to distinct anatomical zones, thereby simulating a lobe-aware screening process.

Consequently, this context-aware initialization provides a strong inductive bias. Instead of starting from scratch, the slots are guided towards informative regions corresponding to the pulmonary lobes from the very first iteration. This “warm-start” mechanism substantially stabilizes the training process and helps the model distinguish between the structural abnormalities of active TB and the stable scars of inactive TB by associating them with their likely anatomical locations.

#### 3.2.2. Iterative Slot Attention with Gated MLP

Once the slots S are initialized, they interact with the dense image features F through an iterative attention mechanism to refine their representations. In this phase, the slots function as diagnostic probes that actively scan the feature map for relevant information. The interaction is mathematically modeled using an attention mechanism where the slots act as queries (Q), and the flattened image features from the backbone act as both keys (K) and values (V). This structure allows the slots to aggregate information from spatial regions that are semantically related to their initialized anatomical roles.

In each iteration, the inputs are projected into distinct subspaces using learnable linear matrices. The query projection transforms the slots to define “what to look for,” while the key and value projections transform the image features to provide the matching criteria and the actual semantic content. The iterative attention update is mathematically formalized in Equation (4). The attention weight matrix A ∈ $R^{B\times N\times L}$ is computed by applying the Softmax function over the spatial dimension L:

(4) $$A=Softmax(\frac{QK^{T}}{\sqrt{D}})$$

This operation generates an attention map which quantifies the relevance of each spatial token in the image to each of the five lobe-specific slots. Following the attention update, the aggregated information is processed by a Gated MLP block, specifically using the SwiGLU activation function [50]. This block replaces the standard feed-forward networks typically found in original transformer architectures. The choice of SwiGLU is motivated by its superior performance in modeling complex non-linear relationships compared to standard activations. The SwiGLU block is mathematically defined in Equation (5):

(5) $$SwiGLU\left(x\right)=\left(xW_{1}\right)⊗SiLU(xW_{2})$$

where $W_{1}$ and $W_{2}$ represent learnable weight matrices, SiLU [51] denotes the activation function, and ⊗ denotes element-wise multiplication. This formulation effectively creates a gating mechanism that controls the flow of information.

This specialized gating structure acts as a noise filter. In the context of TB screening, many regions of the X-ray contain irrelevant information such as healthy tissue, bones, or medical devices. The SwiGLU block allows the model to selectively amplify the signals related to pathological lesions while suppressing the background noise. By multiplying the linear projection x_1_ with the non-linear gate determined by SiLU, the network can sharpen the feature representation, ensuring that the slots retain only the most discriminative features necessary for classification.

Finally, the gated output is projected by a final learnable linear matrix to restore the original feature dimension D and facilitate channel-wise information mixing. This refined slot tensor, now with shape B × N × D, contains the distilled semantic information of the image. Through multiple iterations of this attention-and-gating process, the slots progressively drift from their initial lobe-based anchors to highly specific representations of the actual disease state present in the patient.

### 3.3. Multi-Stage Feature Refinement

As illustrated in Figure 1b, the overall framework adopts a multi-stage residual learning paradigm across three refinement stages: Stage A, Stage B, and Stage C. Inspired by ResNet, identity shortcut connections with element-wise addition are employed within and across these stages to facilitate gradient flow and enable deep hierarchical feature learning without degradation.

Stages A and B share a structural configuration designed for progressive feature abstraction. Each stage initiates with a Squeeze-and-Excitation (SE) block [52], which recalibrates channel-wise responses by compressing spatial dimensions and generating channel weights with a reduction ratio of 16. We maintain the high-dimensional feature embedding of D = 1024 across both refinement stages to prevent the loss of semantic details, avoiding channel reduction. This design choice ensures that fine-grained radiological details are preserved throughout the deep refinement process, while the SE block filters background noise and emphasizes informative channels. Subsequently, the features are processed by the CGSM module, where output slots are aggregated via max pooling and added back to the input features through a residual connection. For a given input feature $F_{in}$, this structural progression in Stages A and B is formalized in Equation (6):

(6) $$F_{out}=F_{in}+MaxPool(CGSM(SELayer(F_{in})))$$

Stage A functions as a preliminary filter to identify coarse lesion patterns, while Stage B deepens this abstraction to capture subtle pathological signs associated with active TB.

Stage C concludes the refinement process by balancing local details with global context. While it retains the SE block for final channel calibration, it excludes the slot attention mechanism. Instead, Stage C fuses the locally refined features from Stage B ($F_{B}$) with the global context vector g derived from the initial backbone features. This residual integration is computed directly via element-wise addition, as shown in Equation (7):

(7) $$F_{final}=SELayer(F_{B})+g$$

This residual integration prevents overfitting to local details and ensures the final feature representation encompasses both discriminative lesion features and the holistic anatomical context.

### 3.4. Hierarchical Classification Head

The fully refined feature map from Stage C is globally averaged to produce a final feature vector F. This vector is concurrently fed into two separate classification heads: a Screening Head and an Activity Head. The Screening Head projects F into 3 class logits corresponding to Normal, Pneumonia, and Tuberculosis (TB). Simultaneously, the Activity Head projects F into a single logit for binary classification (Active vs. Inactive TB). These linear projections are formulated in Equation (8):

(8) $$Z_{scr}={W_{scr}}^{T}F+b_{scr},\;\;Z_{act}={W_{act}}^{T}F+b_{act}$$

Here, the subscripts scr and act explicitly denote the screening and activity tasks, respectively. $W_{scr}$ and $W_{act}$ are the learnable weight matrices, and $b_{scr}$ and $b_{act}$ are the respective bias terms for each task. The predicted probability distribution for the screening task (${\hat{Y}}_{scr}$) is obtained via the Softmax function, while the probability for the activity task (${\hat{y}}_{act}$) is calculated using the Sigmoid function, as shown in Equation (9):

(9) $${\hat{Y}}_{scr,c}=\frac{exp(Z_{src,c})}{\sum_{j=1}^{3}exp(Z_{src,j})},\;\;{\hat{y}}_{act}=\frac{1}{1+exp({-Z}_{act})}$$

In this formulation, the subscript c represents the specific class index for the screening task (e.g., 1 for Normal, 2 for Pneumonia, and 3 for Tuberculosis), and the subscript j serves as the summation index across all three classes. Both heads are trained simultaneously using a multi-task learning objective. For the screening task, we employ Categorical Cross-Entropy (CCE) loss ($L_{scr}$). To prevent overconfidence, label smoothing [53] with a factor of α = 0.1 is applied to the one-hot encoded ground truth $Y_{scr}$. For the activity task, we utilize Binary Cross-Entropy (BCE) loss ($L_{act}$) with the same smoothing factor.

To maintain coherent hierarchical predictions, we adopt a conditional loss masking strategy [54]. The activity classification gradients are backpropagated only for samples identified as TB. This is implemented using an indicator function $1_{TB}$, which equals 1 if the ground truth is TB, and 0 otherwise. The task-specific losses are defined in Equation (10):

(10) $$L_{scr}=-\sum_{c=1}^{3}Y_{scr,c}^{LS}log\left({\hat{Y}}_{scr,c}\right)\;,\;L_{acr}=-1_{TB}[y_{act}^{LS}log({\hat{y}}_{act})+(1-y_{act}^{LS})log(1-{\hat{y}}_{act})]$$

where $Y^{LS}$ and $y^{LS}$ denote the smoothed targets. Furthermore, to effectively balance these two distinct tasks without manual weight tuning, we introduce an Uncertainty-Weighted Loss mechanism [55]. This learnable loss function dynamically adjusts the contribution of each task based on its homoscedastic uncertainty ($\sigma_{1}$, $\sigma_{2}$) during training. The final total loss $L_{total}$ optimized by the network is formulated in Equation (11):

(11) $$L_{total}=\frac{1}{2{\sigma_{1}}^{2}}L_{scr}+\frac{1}{2{\sigma_{2}}^{2}}L_{act}+log(\sigma_{1})+log(\sigma_{2})$$

This mathematical formulation ensures that the model optimizes both screening and activity classification objectives in a balanced manner without letting the activity head learn irrelevant features from non-TB samples.

## 4. Experimental Setup and Evaluation Roadmap

### 4.1. Data Preprocessing and Augmentation Strategy

The original chest radiographs exhibited variable resolutions, averaging approximately 2765 × 2925 pixels for the SCH-CXR dataset and 1389 × 1041 pixels for the kaggle-CXR dataset, and 2109 × 2193 pixels for the DA and DB datasets. All input chest X-ray images were standardized to a resolution of 512 × 512 pixels to balance the retention of fine anatomical details with computational efficiency. Following resizing, we applied min-max normalization to scale pixel intensities to the [0, 1] range. Although direct resizing alters the original aspect ratios, the consistent performance across internal and external evaluations indicates that the proposed framework effectively handles these geometric variations without significant performance degradation. Subsequently, the images were standardized using the mean (0.485, 0.456, 0.406) and standard deviation (0.229, 0.224, 0.225) of the ImageNet dataset [56]. This step is critical for stabilizing the input distribution and facilitating faster convergence when utilizing pre-trained backbones.

To mitigate overfitting and enhance model robustness against diverse imaging conditions, we implemented a customized augmentation pipeline optimized for chest radiography utilizing the Albumentations library [57]. Unlike generic strategies, this pipeline simulates clinical heterogeneity while preserving pathologically significant features. Geometric transformations mimicked patient positioning variations, employing horizontal flipping for data doubling. While this alters cardiac orientation, it preserves the morphological validity of lung lesions. Shift, scale, and rotation operations were strictly constrained to minor deviations to prevent cropping essential anatomical landmarks like lung apices or costophrenic angles which are crucial for diagnosing apical TB and pleural effusion.

**Figure 2:**
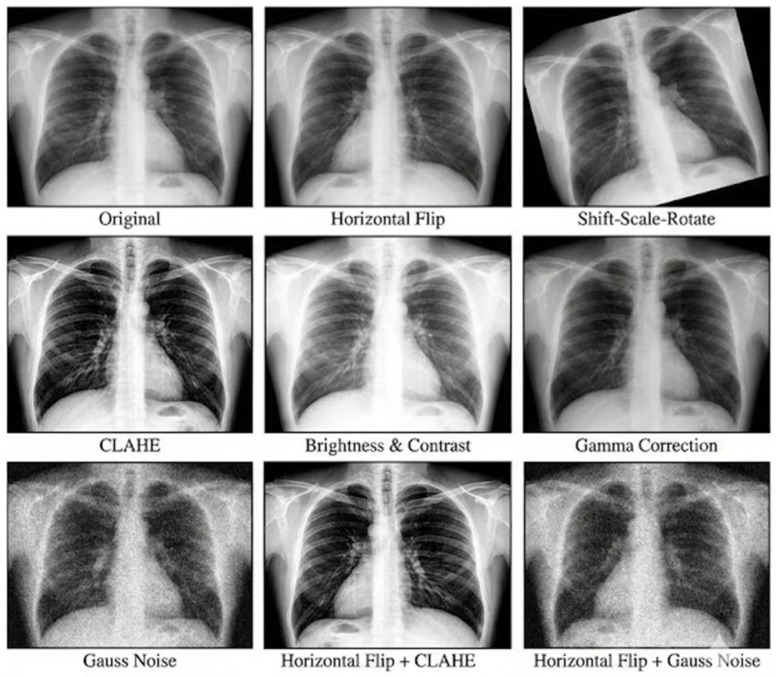
Visualization of data augmentation samples.

We further replicated acquisition protocol variability and sensor noise by applying a stochastic set of intensity and texture transformations inspired by the design philosophy of RandAugment [58]. The strategy randomly selects two transformations from a candidate pool with a high probability. This pool includes Contrast Limited Adaptive Histogram Equalization applied with a conservative clip limit to enhance local contrast, aiding the separation of hilar structures from inactive TB calcifications without generating artifactual nodules. Random Brightness and Gamma simulate radiation dose and exposure time variations with restricted contrast limits to maintain characteristic ground-glass opacity and pneumonia consolidation patterns. Gaussian Noise mimics quantum noise in low-dose scans with low variance to avoid confusing synthetic noise with miliary TB nodules. Additionally, sharpening enhances high-frequency details for delineating fibrotic streaks and cavity walls, while grid distortion models natural anatomical variations in lung shape. This domain-specific augmentation strategy ensures the model learns robust representations invariant to acquisition artifacts while remaining sensitive to subtle pathological signatures, as visually delineated in Figure 2.

### 4.2. Dataset and Ethics Statement

The evaluation phase is structured to systematically validate the proposed method using distinct datasets. The primary evaluation was conducted using a retrospective clinical dataset collected from our institution, hereafter referred to as the SCH-CXR dataset. This dataset serves as the basis for model training, internal evaluation, and stability analysis. To further assess the generalization capability across different data distributions and acquisition protocols, we incorporated publicly available external datasets, specifically the multi-class Kaggle-CXR dataset and the binary DA and DB dataset.

#### 4.2.1. SCH-CXR Dataset

The primary dataset utilized in this study was retrospectively collected from patients who underwent chest X-ray examinations at Soonchunhyang University Cheonan Hospital. The data collection encompassed cases acquired prior to the study approval date, ensuring a comprehensive representation of clinical scenarios. The study protocol strictly adhered to the ethical guidelines outlined in the Declaration of Helsinki and received approval from the Institutional Review Board (IRB) of the hospital under Approval No. SCHCA 2025-06-025.

To ensure patient privacy, all DICOM metadata were stripped, and images were provided in de-identified PNG format. The diagnostic labels were organized through a hierarchical directory structure: Normal, Pneumonia, and Tuberculosis, with the latter subdivided into Active and Inactive TB. Active TB was defined as culture-confirmed pulmonary tuberculosis cases. Inactive TB cases were defined as patients with radiological evidence of prior TB sequelae (e.g., fibrotic scarring, calcified granulomas, or volume loss) but negative microbiological test results and no clinical indication for anti-tuberculosis treatment. The hierarchical classification was grounded on microbiological confirmation, ensuring that the activity prediction task reflects real-world clinical decision-making rather than purely radiological pattern recognition. Consequently, demographic information was unavailable for analysis. Images containing significant foreign bodies, severe artifacts, or ambiguous labels were excluded to ensure data quality.

The dataset was partitioned into training, validation, and test subsets at a ratio of 80:10:10 (80% for training, 10% for validation, and 10% for testing) using stratified random splitting. This stratification maintains consistent class distribution across all subsets, as summarized in Table 1. While the training and validation sets were strictly utilized for model optimization and hyperparameter tuning to prevent overfitting, the independent test set served as the sole standard for the final performance evaluation reported in this study. Additionally, 5-fold cross-validation was applied separately to analyze the stability of the proposed model.

#### 4.2.2. Generalization Analysis for Open Dataset

To evaluate the generalization capability across different data distributions, we utilized two external datasets. First, the Kaggle-CXR dataset [59] comprises four classes: Normal, Pneumonia, COVID-19, and Tuberculosis. As summarized in Table 2, we merged the validation samples into the test set to ensure a statistically meaningful evaluation. To align with our hierarchical architecture, Bacterial Pneumonia and COVID-19 were grouped into a single acute inflammatory super-class.

Second, to explicitly assess binary classification capability, we incorporated the DA and DB Tuberculosis Chest X-ray Dataset [60]. Sourced from the National Institute of Tuberculosis and Respiratory Diseases, this dataset contains 278 frontal chest X-rays. As detailed in Table 2, it is partitioned into a training set and a balanced test set. This independent clinical source enables a targeted evaluation of the model’s ability to distinguish Normal from Tuberculosis.

### 4.3. Implementation Details

The proposed Vision Mamba CGSM framework was implemented using Python 3.8.13 and PyTorch 1.13.1 [61]. All experiments were conducted on a computing server running the Ubuntu 22.05 operating system. The hardware configuration consisted of an Intel Gold 622R CPU, 250 GB of RAM, and four NVIDIA A100 GPUs. We employed the VisionMamba-Base model, pre-trained on the ImageNet-1K dataset, as the backbone network with an embedding dimension of 1024. To optimize memory usage and computational throughput, we enabled Automatic Mixed Precision training utilizing float16 precision. The network was trained for a total of 100 epochs with a physical batch size of 104. To ensure reproducibility, we fixed random seeds for data loading and weight initialization.

All models were trained using Gradient Accumulation over 10 steps, maintaining a consistent effective batch size of 1040. The model parameters were optimized using the AdamW optimizer [62] with a decoupled weight decay of 0.01. Specifically, we disabled weight decay for bias terms, normalization layers, and specific SSM parameters namely the state transition evolution, the input pass-through D, and the timescale discretization ${dt}_{bias}$ to preserve the sequential dependencies and prevent the underfitting of these sensitive components. The learning rate was set to 1 × 10^−4^ for both the backbone and classification heads, managed by a scheduler that combines a linear warmup for the first 10 epochs with a cosine annealing strategy for the remaining iterations.

To address class imbalance, we employed the Weighted Random Sampler in PyTorch with replacement, assigning weights of 2.0 to Normal and Pneumonia classes and 1.0 to each Tuberculosis sub-class, achieving a balanced 1:1:1:1 distribution across all categories. The model was optimized using the Uncertainty-Weighted loss strategy described in Section 3.4 with a label smoothing factor of 0.1. Detailed hyperparameters are summarized in Table 3.

### 4.4. Evaluation Metrics

To evaluate the diagnostic performance of the proposed framework, standard classification metrics derived from the confusion matrix were utilized. For the multi-class screening task, we put emphasis on average metrics to solve class imbalance, while we used standard binary metrics for the binary activity classification task.

The model performance is evaluated using Sensitivity, Specificity, and Accuracy. As defined in Equation (12), Sensitivity measures the ability to correctly identify positive cases, which is critical for minimizing missed diagnoses. Specificity, formulated in Equation (13), assesses the capability to correctly reject negative cases, essential for reducing false alarms. These metrics are mathematically defined as follows:

(12) $$Sensitivity=\frac{1}{K}\sum_{i=1}^{K}\frac{{TP}_{i}}{{TP}_{i}+{FN}_{i}}$$

(13) $$Specificity=\frac{1}{K}\sum_{i=1}^{K}\frac{{TP}_{i}}{{TN}_{i}+{FP}_{i}}$$

Additionally, we report the Accuracy, as shown in Equation (14), which averages the individual per-class accuracy. This formulation prevents the overall performance from being skewed by majority classes, ensuring a fair assessment across all diagnostic categories, providing a robust macro-metric of performance specifically for handling imbalanced datasets.

(14) $$Accuracy=\frac{1}{K}\sum_{i=1}^{K}\frac{{TP}_{i}+{TN}_{i}}{{TP}_{i}+{TN}_{i}+{FP}_{i}+{FN}_{i}}$$

where K denotes the number of classes, and ${TP}_{i}$, ${TN}_{i}$, ${FP}_{i}$, and ${FN}_{i}$ represent the True Positives, True Negatives, False Positives, and False Negatives for the i-th class, respectively. Finally, for model selection, we utilized Youden’s Index (J) [63]. As defined in Equation (15), this metric captures the discriminative power by synthesizing sensitivity and specificity into a single statistic:

(15) J=Sensitivity+Specificity−1

Model selection was governed by the Youden Index calculated on the validation set to identify the checkpoint exhibiting the most favorable trade-off between sensitivity and specificity. This approach aligns the model’s performance with clinical screening requirements where minimizing false negatives is paramount. thereby optimizing the decision threshold for reliable diagnosis. Additionally, Receiver Operating Characteristic (ROC) curves and the Area Under the Curve (AUC) were generated to visually and quantitatively evaluate the diagnostic capacity across various decision thresholds for all tested datasets.

## 5. Experimental Results

In this section, we present a comprehensive evaluation of the proposed Vision Mamba CGSM framework. The evaluation is systematically structured according to the employed datasets. Section 5.1 details the internal validation and statistical stability using the primary SCH-CXR dataset. Section 5.2 and Section 5.3 investigate cross-dataset generalization using the Kaggle-CXR and DA and DB datasets, respectively. Section 5.4 provides visual interpretations using Grad-CAM, and Section 5.5 presents an ablation study on the hierarchical head architecture.

### 5.1. Performance Measurement on SCH-CXR Dataset

We first evaluated the generalization capability of the proposed framework on the independent test set of the primary SCH-CXR dataset. Table 4 presents the quantitative results across the hierarchical diagnostic tasks. This evaluation is critical for assessing how well the learned features, specifically the lobe-aware representations handled by the CGSM, transfer to unseen data distributions. Table 4 presents the quantitative results across the hierarchical diagnostic tasks. Consistent with the validation analysis, the Youden Index (J) serves as the principal metric for determining the overall effectiveness of the models, supplemented by accuracy and task-specific metrics.

As summarized in Table 4, the proposed Vision Mamba CGSM demonstrated significant generalization capability, achieving state-of-the-art performance across all hierarchical levels in terms of the Youden Index (J). In the overall 4-class classification task, our model outperformed all comparative architectures, recording the highest J of 79.55%, Accuracy of 92.96%, Sensitivity of 84.94%, and Specificity of 94.61%. This performance advantage over both the high-capacity CNN baseline EfficientNet-B7 with J = 78.43% and the SSM baseline Vim with J = 79.40% confirms that the dual-path refinement strategy effectively captures stable feature representations that are not prone to overfitting. Similarly, in the 3-class screening task, our framework maintained its leadership with the highest J of 77.67% and Specificity of 92.97%, reinforcing its reliability as a first-line screening tool that minimizes false alarms.

In the clinically critical binary activity classification task between Active and Inactive TB, the proposed model demonstrated the most consistent performance balance. While ResNet152 achieved the highest Sensitivity of 94.62% at the cost of lower Specificity of 95.94%, and ConvNeXt achieved the highest Specificity of 97.15% but suffered from poor Sensitivity of 91.51%, our framework achieved an optimal trade-off. The Vision Mamba CGSM achieved the highest Binary Youden Index of 90.83% and Accuracy of 95.91% among all models. Although its Sensitivity of 93.79% was slightly lower than that of ResNet152, it maintained a significantly higher Specificity of 97.04%. This result suggests that the high-dimensional feature preservation and slot-based attention mechanism allow the model to distinguish active lesions from inactive scars with greater precision than legacy CNNs, effectively mitigating the trade-off between missing active cases and over-diagnosing inactive ones.

**Figure 3:**
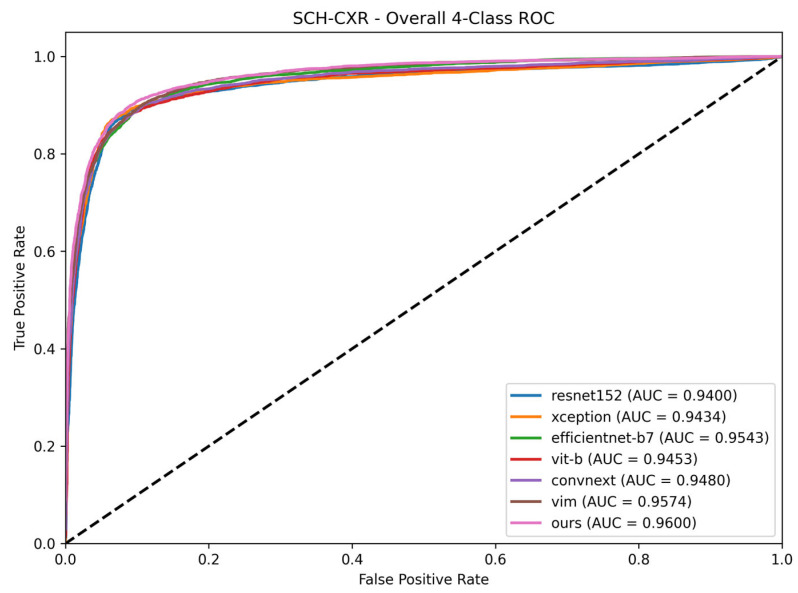
Receiver Operating Characteristic (ROC) curves of the evaluated models on the SCH-CXR test set. The Area Under the Curve (AUC) is provided for each architecture to quantitatively compare the trade-off between sensitivity and specificity. The dashed diagonal line represents the performance of a random classifier (AUC = 0.5).

To visually confirm these results, the ROC curves for the internal SCH-CXR test set are presented in Figure 3. The proposed Vision Mamba CGSM maintains a higher AUC compared to the baseline architecture, indicating stable discriminative capabilities across all diagnostic thresholds.

It is worth noting that the proposed Vision Mamba CGSM requires a relatively higher number of parameters (144.27 M) compared to traditional baseline models such as Xception (20.82 M) or ResNet152 (58.18 M). However, this increase in model capacity is a direct consequence of integrating the Context-Guided Slot Mixer and the dual-path refinement architecture, which are essential for capturing both global context and fine-grained pathological anomalies. Given the critical nature of tuberculosis screening, where misclassification can lead to severe public health consequences or unnecessary treatments, the substantial improvements in the Youden Index and diagnostic robustness fully justify the added structural complexity.

To verify the statistical stability of the framework, we subsequently conducted a 5-fold cross-validation. Table 5 summarizes the mean performance and standard deviation for each diagnostic task across the five folds. The entire dataset was partitioned into five mutually exclusive folds, and the training and evaluation process was repeated five times, ensuring that every sample served as validation data exactly once. This systematic validation process allows us to assess the variance in model performance and confirm its reliability across different subsets of the patient population. Table 5 summarizes the mean performance and standard deviation for each diagnostic task across the five folds.

As shown in Table 5, the proposed Vision Mamba CGSM exhibited high stability across all folds. For the primary 4-class classification task, the model achieved a mean Youden Index of 79.65% ± 0.44% and a mean accuracy of 93.01% ± 0.22%. The extremely low standard deviation of less than 0.5% for the Youden Index indicates that the discriminative power of the model is consistent and robust to variations in data distribution. It is worth noting that the mean cross-validation Youden Index of 79.65% aligns closely with the performance observed on the independent test set, which was 79.55% as reported in Section 5.1, reinforcing the conclusion that the model generalizes well to unseen data without overfitting to a specific validation split.

In the sub-tasks, the model maintained this high degree of reliability. The 3-class screening task yielded a mean accuracy of 91.35% ± 0.26% and a specificity of 93.11% ± 0.17%, further validating its effectiveness as a consistent screening tool. Similarly, in the binary activity classification, the model demonstrated a mean accuracy of 94.71% ± 0.30%, with sensitivity and specificity remaining balanced across all folds. These results confirm that the integration of the CGSM module and the dual-path refinement strategy provides a stable learning mechanism, making the proposed framework clinically reliable for diverse patient scenarios.

**Figure 4:**
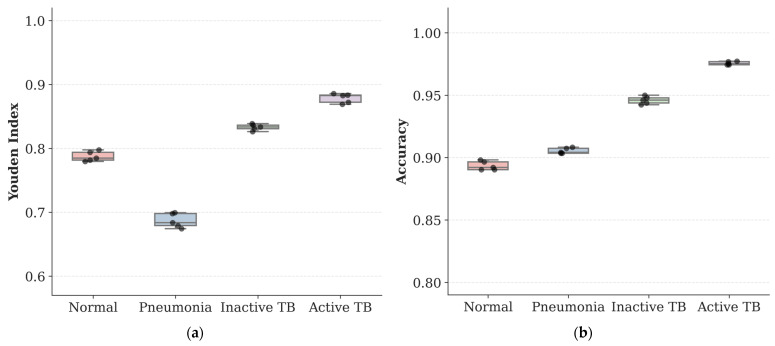

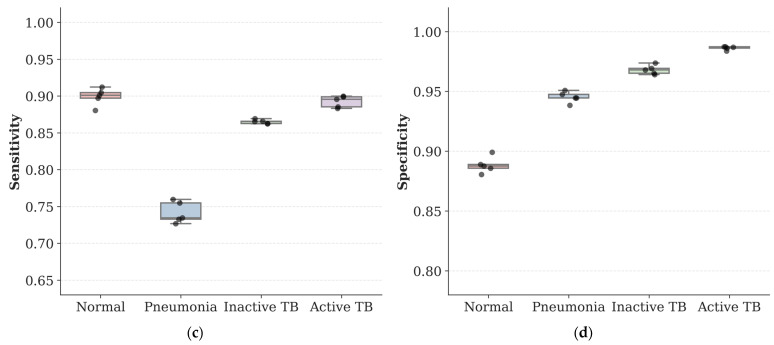
Box plots showing the performance distribution on the SCH-CXR dataset across 5-fold cross-validation. Individual data points are overlaid for each diagnostic class: (**a**) Youden Index; (**b**) Accuracy; (**c**) Sensitivity; and (**d**) Specificity. The central box represents the interquartile range (IQR), the horizontal line indicates the median, and black dots correspond to individual fold results. Y-axis scales are adjusted to highlight distribution variance.

To further investigate the class-wise stability within the overall 4-class classification task, we analyzed the performance distribution for each category including Normal, Pneumonia, Inactive TB, and Active TB. Table 6 presents the detailed statistical breakdown for each class, and Figure 4 visualizes the variance of key metrics using box plots. As illustrated in Figure 4, the interquartile ranges for the Youden Index and accuracy are compactly clustered across all classes, visually corroborating the low standard deviations reported in the overall metrics.

Most importantly, for the clinically critical Active TB class, the model achieved a mean sensitivity of approximately 89.25% and a mean specificity of over 98.63%, with minimal variance across folds. This stability is particularly important for infection control, as it ensures that the high detection rate of infectious cases is reproducible regardless of the data subset. While the Pneumonia class exhibited a relatively lower sensitivity of approximately 74.17% compared to other categories, likely due to radiological overlaps with other consolidations, it maintained a high specificity of over 94.51%. This suggests that the model minimizes false-positive errors even for difficult classes. The Inactive TB and Normal classes also exhibited stable performance metrics, confirming that the proposed framework maintains robust diagnostic capability across the entire hierarchical spectrum without biasing its learning toward specific majority classes.

### 5.2. Performance Measurement on Kaggle-CXR Dataset

To assess generalization across different distributions, we evaluated the model on the multi-class Kaggle-CXR dataset. Table 7 presents the overall classification performance across the four classes (Normal, Tuberculosis, Bacterial Pneumonia, and COVID-19). We compared our method against state-of-the-art baselines. These included CNN-based architectures such as ResNet152, Xception, EfficientNet-B7, and ConvNeXt, in addition to Transformer and SSM-based models like ViT-B and Vim.

Table 7 presents the overall classification performance across the four classes. Ideally, the repurposed hierarchical head would classify Pneumonia vs. COVID-19. However, preliminary experiments showed limited variance in this sub-task due to dataset saturation. Although the absence of biopsy-confirmed activity labels in the external Kaggle dataset precluded a direct evaluation of the Active vs. Inactive TB distinction, the framework exhibited strong generalization capabilities on the consolidated four-class task. By achieving a Youden Index of 94.04%, the model demonstrated that the Context-Guided Slot Mixing module effectively captures robust and transferable feature representations. This suggests that the learned diagnostic features remain valid across different data distributions, even when the specific downstream tasks are adapted to varying clinical taxonomies. This metric functions as the most rigorous performance indicator as it simultaneously evaluates the ability to distinguish healthy cases, identify chronic tuberculosis, and differentiate acute pneumonia subtypes within a single unified framework.

The proposed framework demonstrated superior generalization capabilities, achieving a Youden Index of 94.04%, a sensitivity of 96.33%, and a specificity of 97.71%. While the overall accuracy of 97.34% was on par with the strong CNN baseline ConvNeXt, our model secured a higher Youden Index, indicating a more balanced trade-off between sensitivity and specificity which is critical for clinical screening. Furthermore, our approach significantly outperformed the standalone Vision Mamba backbone which achieved a Youden Index of 93.47%. This improvement validates that the Context-Guided Slot Mixing module effectively enhances the discriminative power of the SSM backbone on unseen data distributions. Compared to traditional CNNs like ResNet152 and EfficientNet-B7 which yielded Youden Indices of 91.45% and 92.00% respectively, our framework offers a more robust solution for diverse clinical environments.

**Figure 5:**
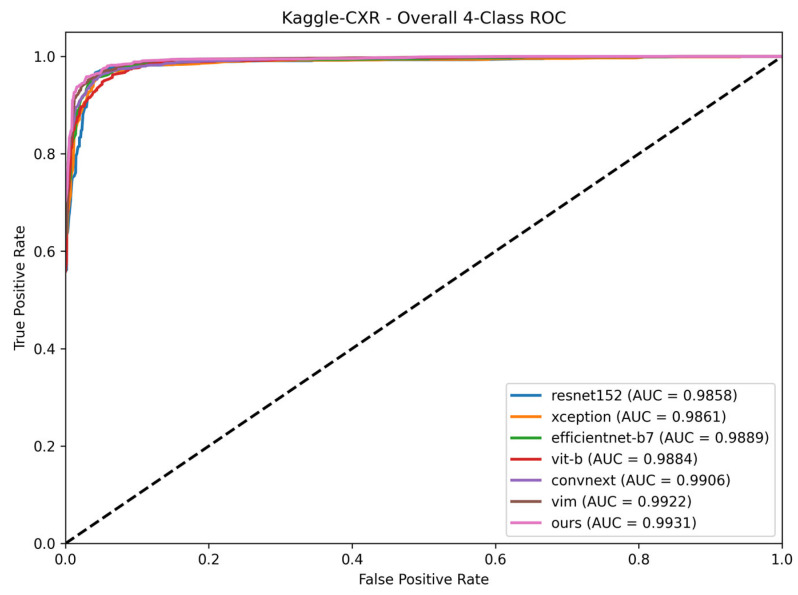
Receiver Operating Characteristic (ROC) curves of the evaluated models on the Kaggle-CXR dataset. The dashed diagonal line represents the performance of a random classifier (AUC = 0.5).

To visually confirm the generalization stability on the multi-class task, the ROC curves for the Kaggle-CXR dataset are presented in Figure 5. The curves illustrate that the proposed framework consistently maintains high AUC scores across diverse disease distributions and acquisition protocols.

### 5.3. Performance Measurement on DA and DB Dataset

To explicitly assess binary classification capability (Normal vs. Tuberculosis) in an external environment, we evaluated the models on the DA and DB dataset. The performance metrics are summarized in Table 8. The proposed Vision Mamba CGSM achieved the highest Youden Index of 80.39% and an overall accuracy of 90.20%. Notably, the model recorded a perfect specificity of 100.00%. While baseline architectures like ConvNeXt and ResNet152 exhibited higher sensitivities (90.20% and 88.24%, respectively), they suffered from lower specificities, indicating a tendency to misclassify normal cases as Tuberculosis. The high specificity of the proposed framework confirms its effectiveness in minimizing false positives, making it reliable for clinical screening. Compared to the standalone Vision Mamba backbone, the integration of the CGSM module improved the Youden Index from 78.43% to 80.39%, demonstrating the practical value of localized feature refinement on completely unseen data.

**Figure 6:**
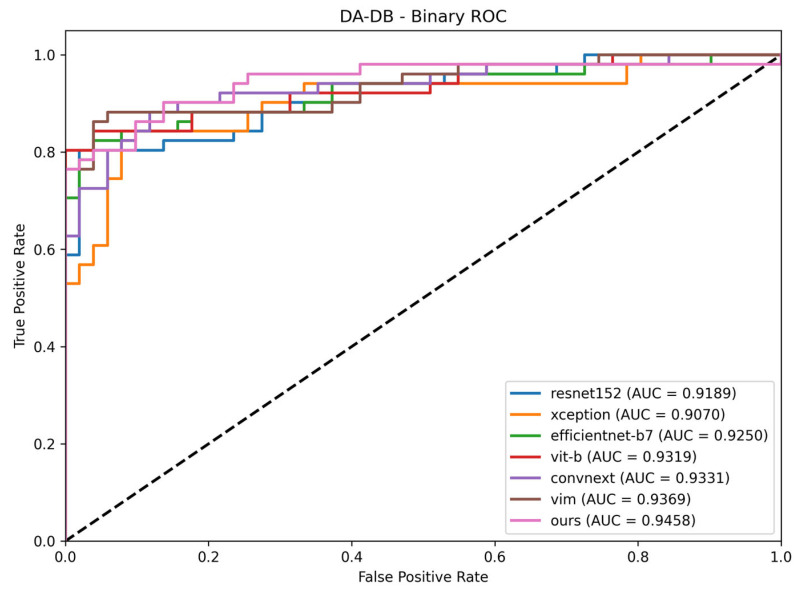
Receiver Operating Characteristic (ROC) curves for binary classification (Normal vs. Tuberculosis) on the DA and DB dataset. The dashed diagonal line represents the performance of a random classifier (AUC = 0.5).

This external generalization performance is further corroborated by the ROC curve analysis shown in Figure 6. The curve verifies the stable discriminative power of the proposed model for the specific binary classification task (Normal vs. Tuberculosis) in a completely distinct clinical setting.

### 5.4. Qualitative Evaluation via Grad-CAM

**Figure 7:**
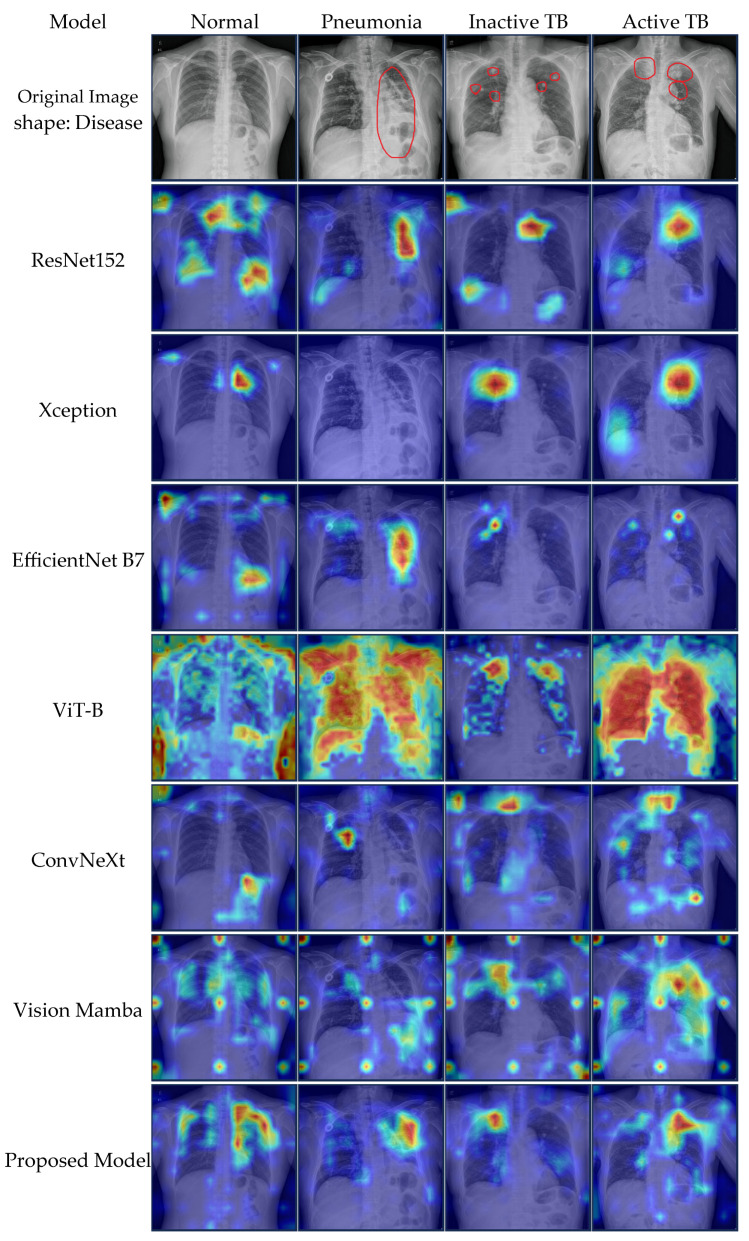
Comparative Grad-CAM visualization results of seven deep learning architectures across four diagnostic classes (Normal, Pneumonia, Inactive TB, and Active TB). The red circles indicate the regions of the lesions (Pneumonia, Inactive TB, and Active TB) explicitly annotated by the medical professionals at the hospital.

To validate the interpretability of the proposed framework, we employed Grad-CAM to visualize the regions of interest (ROI) contributing to the model’s decisions. Figure 7 presents a comparative visualization of the proposed framework against six representative deep learning architectures: ResNet152, Xception [64], EfficientNet-B7, ViT-B, ConvNeXt [65], and Vision Mamba.

The visualization results illustrate the distinct inductive biases inherent in each architectural family. Traditional CNN-based models, specifically ResNet152, Xception, EfficientNet-B7, and ConvNeXt, typically generate smooth and continuous heatmaps. However, due to the aggressive downsampling operations in their pooling layers, these models often produce coarse activation maps that cover broad regions, occasionally failing to capture the fine-grained boundaries of small lesions. In contrast, the Transformer-based ViT-B exhibits characteristic grid-like artifacts with disjointed high-activation patches. This phenomenon results from the non-overlapping patch tokenization strategy, which disrupts the spatial continuity required for precise lesion delineation [66].

Regarding the SSM baselines, Vision Mamba generates fluid and continuous heatmaps, reflecting its ability to model long-range dependencies without patch fragmentation. However, without explicit spatial guidance, Vim tends to produce diffuse activations that occasionally extend into irrelevant background regions, such as the clavicles or the diaphragm, indicating suboptimal spatial localization.

The proposed Vision Mamba CGSM addresses these limitations by integrating the CGSM module. As observed in the final column of Figure 7, our model generates high-contrast heatmaps tightly confined to pathological regions while effectively suppressing the background noise observed in Vim and the coarse activations of CNN. In Pneumonia cases, the model accurately maps the extent of consolidation without fragmentation. For Active TB, the heatmap focuses intensely on core infectious lesions, distinctively separating them from surrounding tissues. This precision extends to Inactive TB, where the model targets fibrotic scarring while avoiding false positive activations in healthy lung fields. These observations indicate that the dual-path refinement strategy facilitates the recalibration of spatial features. The model appears to combine the continuous receptive field of SSMs with the localization precision typically associated with CNNs.

### 5.5. Ablation Study: Impact of Hierarchical Head Architecture

To isolate the structural contribution of the hierarchical design, we conducted an ablation study exclusively on the proposed Vision Mamba CGSM framework. We constructed a baseline by modifying only the head structure of our model while keeping the SSM backbone and feature extraction layers identical. Specifically, we compared the proposed Hierarchical Multi-Head configuration against a Unified Single-Head configuration derived from the same framework. The Unified Single-Head variant utilizes a standard classification layer that treats all classes as a single label vector, whereas our proposed method structurally decouples disease categorization from activity classification. To enable evaluation on the external Kaggle-CXR dataset which lacks explicit activity labels, we mapped the existing four classes into a hierarchical structure compatible with our framework. This adaptation ensured that the multi-head mechanism remained active during the external validation process. The resulting quantitative comparison on both the internal SCH-CXR dataset and the external Kaggle-CXR dataset is presented in Table 9.

On the internal SCH-CXR dataset, the Hierarchical Multi-Head model achieved a Youden Index of 79.55%. This represents a marginal improvement of 0.23% over the Unified Single-Head baseline which scored 79.32%. Sensitivity increased by 0.24%, whereas Specificity remained comparable with a minimal fluctuation of 0.01%. This indicates that the hierarchical structure enhances disease detection capability while effectively preserving the high specificity of the baseline. These results suggest that for the internal distribution, both configurations approach the diagnostic ceiling imposed by the radiological ambiguity of biopsy-confirmed labels.

The advantages of the hierarchical configuration become more prominent in the external generalization task. On the Kaggle-CXR dataset, our method recorded a Youden Index of 94.04%, outperforming the baseline score of 92.85% by 1.19%. Sensitivity markedly increased from 95.41% to 96.33%. While the Unified Single-Head configuration tends to overfit the specific label distribution of the training set, the Hierarchical Multi-Head configuration explicitly separates the learning objectives. This separation enforces the extraction of more robust and generalized features. This structural benefit is achieved with negligible additional computational cost, as the added projection layer comprises a minimal fraction of the total parameters. Thus, the proposed head design is critical for ensuring generalization performance beyond internal validation.

## 6. Discussion

In this study, we presented a hierarchical deep learning framework utilizing the State Space Model architecture to enhance both diagnostic accuracy and interpretability in chest X-ray analysis. The experimental results confirm that our proposed Vision Mamba CGSM outperforms existing state-of-the-art models, particularly in the challenging task of differentiating Active from Inactive Tuberculosis. In this section, we interpret these findings from multiple perspectives: the structural advantages of the proposed module, the clinical implications of the performance metrics, the visual explainability of the model, and the limitations that need to be addressed in future research.

### 6.1. Architectural Effectiveness of SSM and CGSM

The primary contribution of this study is the successful integration of the Mamba-based SSM with the proposed CGSM module. Traditional CNNs such as EfficientNet and ResNet excel at extracting local texture features but struggle with global context due to their limited receptive fields. Conversely, ViTs capture global dependencies but often fail to delineate fine-grained local boundaries due to their patch-based tokenization. Our results in Table 4 demonstrate that Vision Mamba CGSM effectively addresses this limitation. The SSM backbone provides a continuous global receptive field, allowing the model to understand the overall lung geometry. Simultaneously, the CGSM module acts as a spatial attention gate, recalibrating feature maps to emphasize lesion-specific patterns. This integration is evidenced by the superior Youden Index (79.55%), which indicates a balanced optimization of sensitivity and specificity, surpassing both the CNN-based ConvNeXt and the pure SSM-based Vim.

### 6.2. Clinical Utility and Trustworthiness

A critical bottleneck in automated TB screening is the high false-positive rate caused by Inactive TB, characterized by healed scars or fibrosis which mimics the radiological appearance of Active TB. In our experiments, heavy CNNs like ResNet152 achieved high sensitivity but a lower specificity of 95.94%, implying a tendency to over-diagnose inactive scars as active disease. In contrast, our proposed model achieved the highest specificity of 97.04% while maintaining competitive sensitivity. This suggests that the feature preservation capability of our architecture avoiding aggressive pooling and maintaining high-dimensional spatial representations enables the model to detect subtle radiological signs of activity, such as soft consolidations or cavities, while correctly ignoring calcified scars. This characteristic is vital for optimizing clinical workflows by reducing the rate of unnecessary follow-up examinations caused by false positives.

This quantitative performance is further supported by our qualitative analysis of model interpretability. The lack of interpretability in deep learning models often serves as a major barrier to clinical adoption; however, the gradient-based visualization provided in Section 5.2 offers strong evidence of the model’s reliability. While comparative models like ViT-B exhibited grid-like artifacts and the baseline Vim showed diffuse activations covering irrelevant background areas, the Vision Mamba CGSM demonstrated high-contrast, tightly confined activations centered strictly on the core pathological regions. By suppressing background noise and sharpening the focus on lesions, our model offers visual explanations that align well with radiological findings. This alignment between high diagnostic specificity and precise visual localization reinforces the trustworthiness of the system, supporting radiologists in their decision-making process rather than merely providing a binary classification.

### 6.3. Limitations

While this study demonstrates the efficacy of the Vision Mamba CGSM framework, several limitations require acknowledgment to fully contextualize our findings. Addressing these constraints provides a clearer perspective on the current boundaries of the proposed model and identifies necessary directions for future research.

First, regarding data generalization, the SCH-CXR dataset provided a robust training ground for our primary objectives. However, the specific task of Tuberculosis activity classification could not be fully validated on the external Kaggle-CXR dataset. This restriction occurred because most public datasets lack the granular and biopsy-confirmed labels required to distinguish between Active and Inactive TB. Consequently, the validation of our hierarchical component remained confined to internal data resources where such detailed annotations were available.

Second, the current framework relies solely on radiographic imaging analysis. In actual clinical practice, TB diagnosis operates as a multimodal process that incorporates patient history, symptoms, and microbiological test results. Therefore, the model’s diagnostic specificity may face constraints in complex cases where radiological findings appear ambiguous while clinical metadata offers decisive clues. Future iterations would benefit from integrating these non-imaging data points to enhance diagnostic precision.

Third, regarding interpretability, Grad-CAM serves as a post hoc explanation method that highlights correlations rather than strictly causal relationships. Although this technique proves effective for localization, it may not fully capture the complex reasoning mechanisms inherent to the SSM backbone. This discrepancy leaves a potential gap in achieving fully transparent decision-making, particularly concerning the non-local dependencies utilized by the model.

Fourth, cross-validation analysis revealed a relative reduction in sensitivity for the Pneumonia class compared to Tuberculosis categories. This performance disparity primarily stems from the inherent radiological similarities between bacterial pneumonia consolidations and other pulmonary opacities. Such visual overlaps confound the model in the absence of distinct differentiating features, leading to occasional misclassification in this specific category.

Finally, the retrospective design of this study limits the immediate assessment of the model’s real-world impact. Specifically, factors such as radiologist workflow efficiency and long-term patient outcomes remain outside the scope of this analysis. Verification of these practical benefits requires prospective clinical implementation and validation across diverse medical environments.

## 7. Conclusions

In this study, we introduced Vision Mamba CGSM, a deep learning framework designed for the hierarchical diagnosis of Tuberculosis. Our primary methodological contribution is the integration of the linear computational efficiency of State Space Models (SSMs) with the localized spatial attention of the Context-Guided Slot Mixing (CGSM) module, directly addressing the limitations of conventional CNN and Transformer architectures.

Experimental evaluations demonstrate the clinical viability of the proposed method. On the primary internal SCH-CXR dataset, the model achieved a Youden Index of 79.55% and an accuracy of 92.96% in the 4-class classification. Notably, in the critical binary task of distinguishing Active from Inactive TB, it recorded a specificity of 97.04%, significantly reducing false-positive diagnoses compared to baseline CNNs. External generalization tests confirmed its stability, yielding a Youden Index of 94.04% on the multi-class Kaggle-CXR dataset and 80.39% with a perfect specificity of 100.00% on the DA and DB dataset.

Building upon these findings, future research will address current limitations to enhance clinical utility. To overcome the constraints of unimodal imaging, we plan to develop a multi-modal fusion architecture that integrates clinical variables such as sputum smear results and patient history. Furthermore, to improve sensitivity for hard-to-distinguish classes like Pneumonia, we will explore advanced training strategies, including curriculum and contrastive learning, to better separate subtle inter-class features. We will also investigate inherent interpretability methods, such as prototype-based learning, to provide transparent decision pathways for the SSM backbone. Ultimately, we aim to validate the real-world efficacy of this system through multi-center prospective clinical trials.

## Figures and Tables

**Table 1:** Overview of the SCH-CXR dataset (80:10:10 split).

Primary Class	Sub-Category	Training Set	Validation Set	Test Set	Total
Normal	-	16,225	2028	2028	20,281
Pneumonia	-	6658	832	833	8323
Tuberculosis	Inactive TB	7282	910	911	9103
	Active TB	3868	484	483	4835
Total		34,033	4254	4255	42,542

**Table 2:** Overview of the external datasets for generalization analysis.

(a) Kaggle-CXR dataset				
**Primary class**	**Sub-class**	**Train**	**Test**	**Total**
Normal	-	1341	242	1583
Pneumonia	Pneumonia	3875	398	4273
	COVID-19	460	116	576
Tuberculosis	-	650	53	703
Total	-	6326	809	7135
(b) DA and DB Tuberculosis Chest X-ray dataset				
**Class**		**Train**	**Test**	**Total**
Normal		102	51	153
Tuberculosis		74	51	125
Total		176	102	278

**Table 3:** Summary of Hyperparameters.

Parameter	Value
Backbone Network	Vision Mamba Base (Pretrained on ImageNet)
Image Resolution	512 × 512
Batch Size	104 (Physical)/1040 (Effective with Gradient Accumulation)
Total Epochs	100
Optimizer	AdamW (Decoupled Weight Decay 0.01)
Learning Rate	1 × 10^−4^
Learning Rate Scheduler	Linear Warmup (10 Epochs) + Cosine Annealing (90 Epochs)
Loss Function (Screening)	Cross-Entropy Loss (Label Smoothing 0.1)
Loss Function (Activity)	Binary Cross-Entropy Loss (Target Smoothing 0.1)
Classification Loss	Categorical Cross-Entropy (Label Smoothing 0.1)
Multi-Task Strategy	Uncertainty-Weighted Loss + Conditional Masking
Sampling Strategy	Weighted Random Sampler (Balanced 1:1:1:1), Drop Last
Precision	Automatic Mixed Precision (FP16)
Normalization	ImageNet Mean & Std
Data Augmentation	Albumentations (Geometric & Intensity Transforms)

**Table 4:** Comparative generalization performance of the proposed framework against representative deep learning architectures on the independent test set of the SCH-CXR dataset. The best results for each metric are highlighted in bold. (J: Youden Index, Acc: Accuracy, Sen: Sensitivity, Spe: Specificity).

Model	Params	Overall				Screening				Activity			
		J	Acc	Sen	Spe	J	Acc	Sen	Spe	J	Acc	Sen	Spe
ResNet152	58.18 M	77.36%	92.15%	83.28%	94.08%	74.90%	90.18%	82.71%	92.20%	90.56%	95.48%	**94** **.** **62** **%**	95.94%
Xception	20.82 M	78.15%	91.99%	84.00%	94.15%	75.70%	90.04%	83.42%	92.28%	90.14%	95.34%	94.20%	95.94%
EfficientNet B7	63.81 M	78.43%	92.56%	84.13%	94.30%	76.03%	90.79%	83.47%	92.56%	90.51%	95.77%	93.58%	96.93%
ViT-B	86.44 M	78.11%	92.80%	83.69%	94.42%	76.16%	91.01%	83.47%	92.69%	89.17%	95.34%	92.13%	97.04%
ConvNeXt	87.57 M	78.55%	92.43%	84.21%	94.35%	76.52%	90.54%	83.95%	92.56%	88.66%	95.19%	91.51%	**97** **.** **15** **%**
Vision Mamba	97.69 M	79.40%	92.60%	84.87%	94.53%	77.59%	90.77%	84.74%	92.85%	90.58%	95.62%	94.20%	96.38%
Proposed Model	144.27 M	**79** **.** **55** **%**	**92** **.** **96** **%**	**84** **.** **94** **%**	**94** **.** **61** **%**	**77** **.** **67** **%**	**91** **.** **27** **%**	**84** **.** **70** **%**	**92** **.** **97** **%**	**90** **.** **83** **%**	**95** **.** **91** **%**	93.79%	97.04%

**Table 5:** Statistical robustness verification of the proposed framework using 5-fold cross-validation on the SCH-CXR dataset. The results are presented as Mean ± Standard Deviation, demonstrating low variance and high stability across different data partitions. (J: Youden Index, Acc: Accuracy, Sen: Sensitivity, Spe: Specificity).

Fold	Overall				Screening				Activity			
	J	Acc	Sen	Spe	J	Acc	Sen	Spe	J	Acc	Sen	Spe
1	79.67%	93.20%	84.88%	94.79%	78.11%	91.58%	84.89%	93.21%	86.21%	94.51%	88.52%	97.69%
2	79.09%	92.84%	84.50%	94.59%	77.46%	91.15%	84.50%	92.96%	85.84%	94.33%	88.31%	97.53%
3	79.68%	92.75%	85.05%	94.64%	77.95%	91.06%	84.94%	93.01%	87.17%	94.69%	89.97%	97.20%
4	80.30%	93.27%	85.44%	94.86%	78.77%	91.66%	85.43%	93.34%	87.41%	94.98%	89.56%	97.86%
5	79.50%	92.99%	84.89%	94.61%	77.59%	91.32%	84.59%	93.00%	87.67%	95.05%	89.87%	97.80%
Mean ± Std	79.65% ± 0.44%	93.01% ± 0.22%	84.95% ± 0.34%	94.70% ± 0.12%	77.97% ± 0.52%	91.35% ± 0.26%	84.87% ± 0.37%	93.11% ± 0.17%	86.86% ± 0.79%	94.71% ± 0.30%	89.25% ± 0.77%	97.62% ± 0.26%

**Table 6:** Detailed statistical breakdown of performance metrics for each diagnostic class derived from 5-fold cross-validation on the SCH-CXR dataset. (J: Youden Index, Acc: Accuracy, Sen: Sensitivity, Spe: Specificity).

Fold	Class	Overall			
		J	Acc	Sen	Spe
1	Normal	79.77%	89.82%	91.20%	88.57%
	Pneumonia	68.36%	90.82%	73.27%	95.09%
	Inactive TB	83.34%	94.61%	86.54%	96.80%
	Active TB	87.21%	97.53%	88.52%	98.69%
2	Normal	78.47%	89.21%	89.69%	88.77%
	Pneumonia	67.90%	90.34%	73.45%	94.45%
	Inactive TB	83.06%	94.38%	86.55%	96.52%
	Active TB	86.92%	97.44%	88.31%	98.61%
3	Normal	77.96%	89.02%	88.04%	89.91%
	Pneumonia	69.80%	90.34%	75.96%	93.83%
	Inactive TB	82.63%	94.23%	86.22%	96.41%
	Active TB	88.35%	97.43%	89.97%	98.38%
4	Normal	79.36%	89.65%	90.46%	88.90%
	Pneumonia	69.93%	90.74%	75.48%	94.45%
	Inactive TB	83.64%	94.99%	86.27%	97.37%
	Active TB	88.28%	97.68%	89.56%	98.73%
5	Normal	78.14%	89.02%	90.09%	88.05%
	Pneumonia	67.41%	90.42%	72.67%	94.74%
	Inactive TB	83.86%	94.79%	86.92%	96.93%
	Active TB	88.59%	97.72%	89.87%	98.73%
Mean ± Std	Normal	78.74% ± 0.79%	89.34% ± 0.37%	89.90% ± 1.18%	88.84% ± 0.68%
	Pneumonia	68.68% ± 1.13%	90.53% ± 0.23%	74.17% ± 1.46%	94.51% ± 0.46%
	Inactive TB	83.31% ± 0.48%	94.60% ± 0.31%	86.50% ± 0.28%	96.81% ± 0.38%
	Active TB	87.87% ± 0.75%	97.56% ± 0.14%	89.25% ± 0.77%	98.63% ± 0.14%

**Table 7:** Overall 4-class classification performance comparison on the Kaggle-CXR dataset. The best results for each metric are highlighted in bold. (J: Youden Index, Acc: Accuracy, Sen: Sensitivity, Spe: Specificity).

Model	Overall			
	J	Acc	Sen	Spe
ResNet152	91.45%	96.66%	94.59%	96.86%
Xception	91.91%	96.79%	94.74%	97.17%
EfficientNet B7	92.00%	96.85%	94.94%	97.06%
ViT-B	91.93%	96.91%	94.78%	97.15%
ConvNeXt	93.23%	**97** **.** **34** **%**	95.66%	97.57%
Vision Mamba	93.47%	97.03%	95.94%	97.53%
Proposed Model	**94** **.** **04** **%**	**97** **.** **34** **%**	**96** **.** **33** **%**	**97** **.** **71** **%**

**Table 8:** Binary classification performance (Normal vs. Tuberculosis) on the external DA and DB dataset. The best results for each metric are highlighted in bold. (J: Youden Index, Acc: Accuracy, Sen: Sensitivity, Spe: Specificity).

Model	Binary			
	J	Acc	Sen	Spe
ResNet152	72.55%	86.27%	88.24%	84.31%
Xception	74.50%	87.25%	82.35%	92.16%
EfficientNet B7	76.47%	88.24%	78.43%	98.04%
ViT-B	76.47%	88.24%	84.31%	92.16%
ConvNeXt	76.47%	88.24%	**90.20%**	86.27%
Vision Mamba	78.43%	89.22%	82.35%	96.08%
Proposed Model	**80.39%**	**9** **0.20%**	80.39%	**100.00%**

**Table 9:** Performance comparison based on Head Configuration. The best results for each metric are highlighted in bold. (J: Youden Index, Acc: Accuracy, Sen: Sensitivity, Spe: Specificity).

Dataset	Head Configuration	Overall			
		J	Acc	Sen	Spe
SCH-CXR	Unified Single-Head	79.32%	92.91%	84.70%	**94.62%**
	Hierarchical Multi-Head (Ours)	**79.55%**	**92.96%**	**84.94%**	94.61%
Kaggle-CXR	Unified Single-Head	92.85%	97.22%	95.41%	97.44%
	Hierarchical Multi-Head (Ours)	**9** **4.04%**	**9** **7.34%**	**9** **6.33%**	**97** **.71%**

## Data Availability

Publicly available datasets were analyzed in this study. This data can be found here: [59,60]. The remaining data presented in this study are not publicly available due to patient privacy and institutional ethics restrictions.
